# Psychometric properties of technology-assisted matching paradigms in post-stroke upper limb proprioceptive assessment: a scoping review

**DOI:** 10.3389/fneur.2025.1556111

**Published:** 2025-06-26

**Authors:** Guiyi Gu, Ruixuan Lin, Roy Rongyue Zeng, Tiev Miller, Jack Jiaqi Zhang

**Affiliations:** ^1^Department of Rehabilitation Sciences, The Hong Kong Polytechnic University, Hong Kong, Hong Kong SAR, China; ^2^Department of Rehabilitation, Dongguan People’s Hospital, Dongguan, Guangdong, China

**Keywords:** assessment, proprioception, psychometrics, rehabilitation engineering, sensory disorders, stroke

## Abstract

**Introduction:**

Proprioceptive impairments affect 34–64% of post-stroke patients, impacting motor recovery and daily activities. Technology-assisted matching paradigms offer precise, quantitative assessment of upper limb proprioception, but their psychometric properties require evaluation.

**Methods:**

The search was conducted using PubMed, Web of Science, EMBASE, and MEDLINE to identify studies on technology-assisted matching paradigms for assessing upper limb proprioception in post-stroke patients. Studies were selected based on the inclusion and exclusion criteria, and relevant data were extracted.

**Results:**

A total of 13 articles were included. Upper limb robots for active mirror-matching tasks were the most used technology among our included studies (9 out of 13 studies). Seven studies showed a moderate level of concurrent validity, and four studies showed a moderate level of convergent validity. Seven studies compared stroke patients to healthy individuals, with most showing good responsiveness. Five studies revealed moderate to high test–retest and inter-rater reliability.

**Conclusion:**

Technology-assisted matching paradigms demonstrate moderate validity and moderate to high reliability when applied in clinical settings for assessing upper limb proprioception in post-stroke patients.

## 1 Introduction

Proprioception is defined as the sense of motion and body position, which enables individuals to have control over their physical orientation (1). Common aspects of proprioception include position sense and kinesthesia (2). Position sense refers to the awareness of body position even at rest (2). Kinesthesia is the ability to perceive the position, movement speed, and direction of one’s limbs during movement (3, 4). Research has shown that approximately 34 to 64% of post-stroke patients experience proprioceptive impairments (5). The impairment of proprioception affects motor recovery (6–8) and independence in performing activities of daily living after stroke (9).

Clinical assessments, such as the Thumb Localizing Test (TLT) (10) and the kinesthetic Up-Down Test (kUDT) (11), have been commonly used for assessing proprioception in post-stroke patients. However, these assessments use ordinal scales with low sensitivity and a noticeable ceiling effect (12). In addition, these assessments rely on the judgment and experience of the assessor, making them susceptible to operator bias (10, 13). In fact, these clinical tests exhibit considerable variability (12) and low test–retest and inter-rater reliability (14, 15). The technology-assisted paradigm has emerged as a promising approach for assessing proprioception. Proprioceptive evaluation typically employs three paradigms: (1) adjusting a stimulus to match a reference, (2) comparing paired stimuli, and (3) detecting the onset or cessation of passive motion. Among these, the matching paradigm is the most widely used (16). In this paradigm, participants actively or passively replicate a target limb position, engaging sensory input, integration, and motor output to identify proprioceptive deficits. The matching paradigm has demonstrated strong reliability and validity, making it a common tool in post-stroke rehabilitation (17). However, existing reviews on proprioceptive assessment often broadly examine all available methods rather than focusing on specific paradigms (18). To address this gap, our review specifically explores the technology-assisted matching paradigm.

Matching paradigm assessments based on various technologies have been developed to quantitatively assess proprioception in healthy and neurological populations (18). Technology-assisted methods, in general, do not rely on subjective observation, and they also can deliver precise and reproducible stimuli (19). These methods also ensure results can be quantified using continuous and norm-based measures (16, 20). Consequently, there are no floor or ceiling effects, allowing for a better evaluation of severity and treatment progress. The matching paradigm, an extensively studied method in research literature, involves moving the limb of the subject to a target position, after which the participant is required to align the contralateral or ipsilateral limb with that target position, either actively or passively (18, 21). This paradigm is potentially time-efficient and suitable for clinical settings (22). To assess kinesthetic sense, the participant’s limb may be moved at a specific speed or through a defined trajectory. The participant is then required to replicate the same movement with the contralateral limb. However, different methods may introduce potential confounders that affect the accuracy of proprioception assessment results (21).

Currently, there is no systematic review evaluating proprioception assessment using the matching paradigm, particularly in poststroke populations. This gap highlights the need for further investigation. Therefore, this scoping review aims to: (1) summarize the use of technology-assisted matching paradigms in the assessment of upper limb proprioception post-stroke and evaluate their psychometric properties; (2) discuss the limitations of current proprioceptive assessments and potential factors influencing assessment outcomes in people with stroke; and (3) determine future research needs in order to design more comprehensive assessment protocols for this population.

## 2 Methods

### 2.1 Searching strategy

To systematically evaluate and review methods for assessing upper limb proprioception in post-stroke patients using new technologies based on the matching paradigm, we conducted searches using four databases: PubMed, Web of Science, EMBASE, and MEDLINE. The following search strategy was employed: (proprioception OR position sense OR kinesthetic sense OR kinesthetics OR position matching OR kinesthetic matching) AND (stroke OR cerebrovascular accident OR cerebral infarction OR cerebral hemorrhage) AND (upper extremity OR upper limb OR arm OR forearm OR shoulder OR elbow OR wrist OR hand OR finger). Each search was conducted from database inception to March 5, 2024.

### 2.2 Inclusion and exclusion criteria

Studies exploring the psychometric properties of proprioceptive assessment tools based on the matching paradigm and utilizing technology assistance (e.g., robots, motion sensors, etc) in adult post-stroke patients (age > 18 years old) were included. To ensure the methodological rigor and reliability of the findings, studies with fewer than five participants were excluded, as such small sample sizes are unlikely to provide sufficient statistical power (23). Reviews, expert opinions, non-English literature, and studies involving perinatal stroke patients were also excluded. Two authors (GG and RL) independently scanned the titles, read the abstracts, identified relevant studies and finalized the list of included studies, according to the inclusion and exclusion criteria. Any discrepancy was resolved by the senior author (JZ).

### 2.3 Data extraction

Data extraction included basic information on subjects, assessment methods, and the psychometric properties of the assessments. Two authors (GG and RL) performed the data extraction independently and discussed with the senior author (JZ), if there was any disagreement.

The information on assessment methods includes the type of proprioception, the equipment used, the body location of the assessment, the matching target and test limb, the type of matching approach, the characteristics of the participants, and the outcome measures.

The types of psychometric properties included in the data extraction were validity, reliability, and responsiveness. Validity refers to the degree to which a test or assessment accurately measures what it claims to measure (24). The types of validity extracted in this review—concurrent, convergent, and divergent—are summarized in Supplementary Table S1, with examples of comparisons drawn from the included studies. Concurrent validity was assessed using established clinical tests as reference measures, despite their known limitations.

The types of reliability included in the review are as follows:

- *Test–retest reliability*: This involves testing the same subjects on two or more separate occasions (25).
- *Inter-rater reliability*: This is agreement between different raters who measure the same group of participants (25).
- *Internal consistency*: This refers to the extent to which the items of a scale or instrument measure various aspects of the same characteristic and nothing else (25).

Responsiveness refers to the sensitivity of measurement (26). We defined two approaches of assessing responsiveness: (1) Area Under Curve (AUC): The area under the Receiver Operating Characteristic (ROC) curve is used to evaluate the ability of an assessment to detect proprioception errors in differentiating stroke patients from healthy control subjects (26). An AUC equal to or greater than 0.70 is regarded as a satisfactory index of responsiveness (27). (2) Discrimination: Compare the target group’s measurements with the normal population using statistical analysis for significant differences (26).

### 2.4 Quality assessment

The quality of the included studies was assessed using the QUADAS-2 tool, which evaluates risk of bias and applicability concerns in diagnostic accuracy studies. The QUADAS-2 tool examines four domains: patient selection, index test, reference standard, and flow and timing. Two reviewers independently assessed each study (GG and RL), and discrepancies were resolved through discussion with the senior author (JZ). The risk of bias judgments for each domain were rated as either “low,” “high,” or “unclear.”

## 3 Results

### 3.1 Searching result

**Figure 1:**
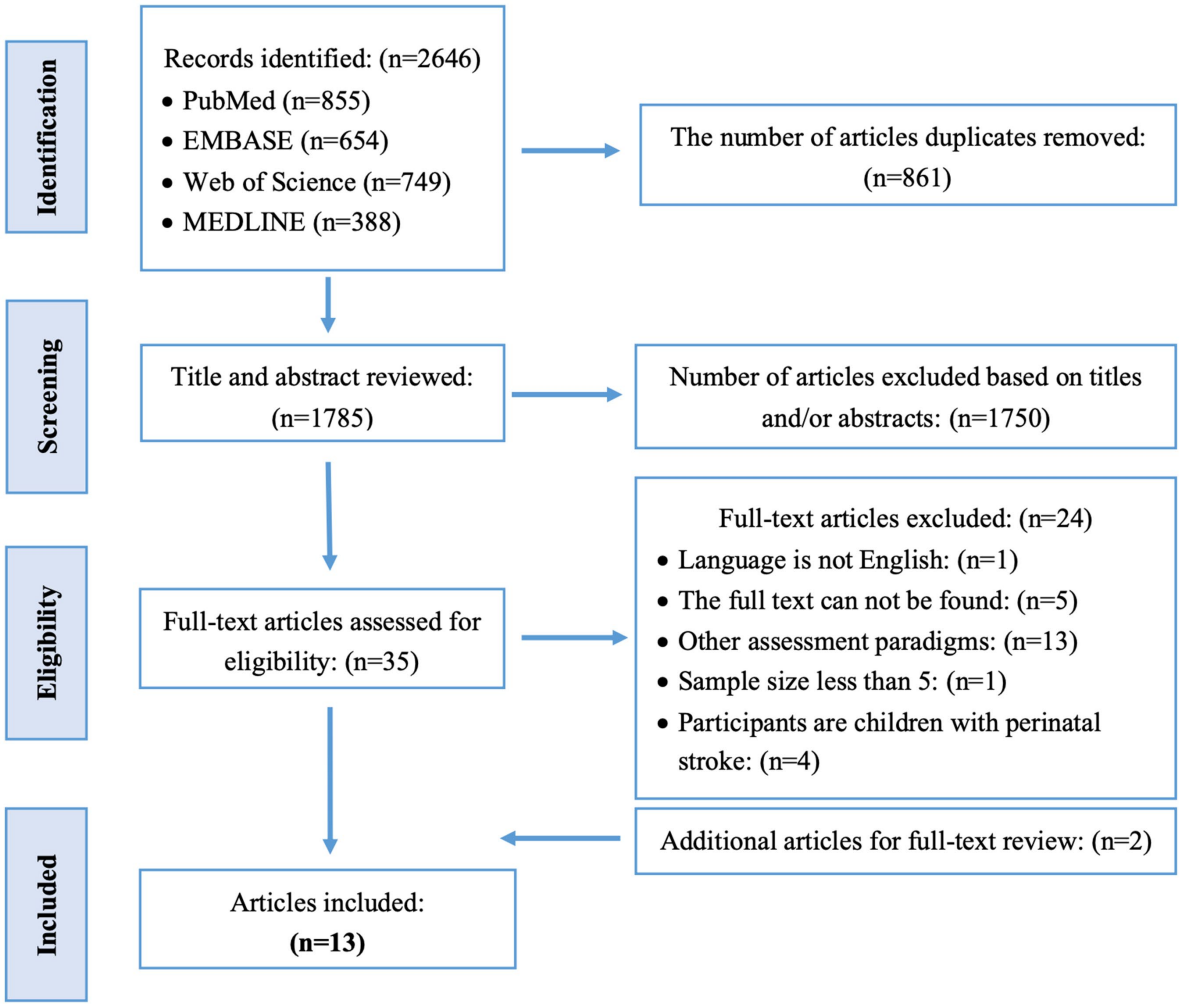
Article selection process.

**Figure 2:**
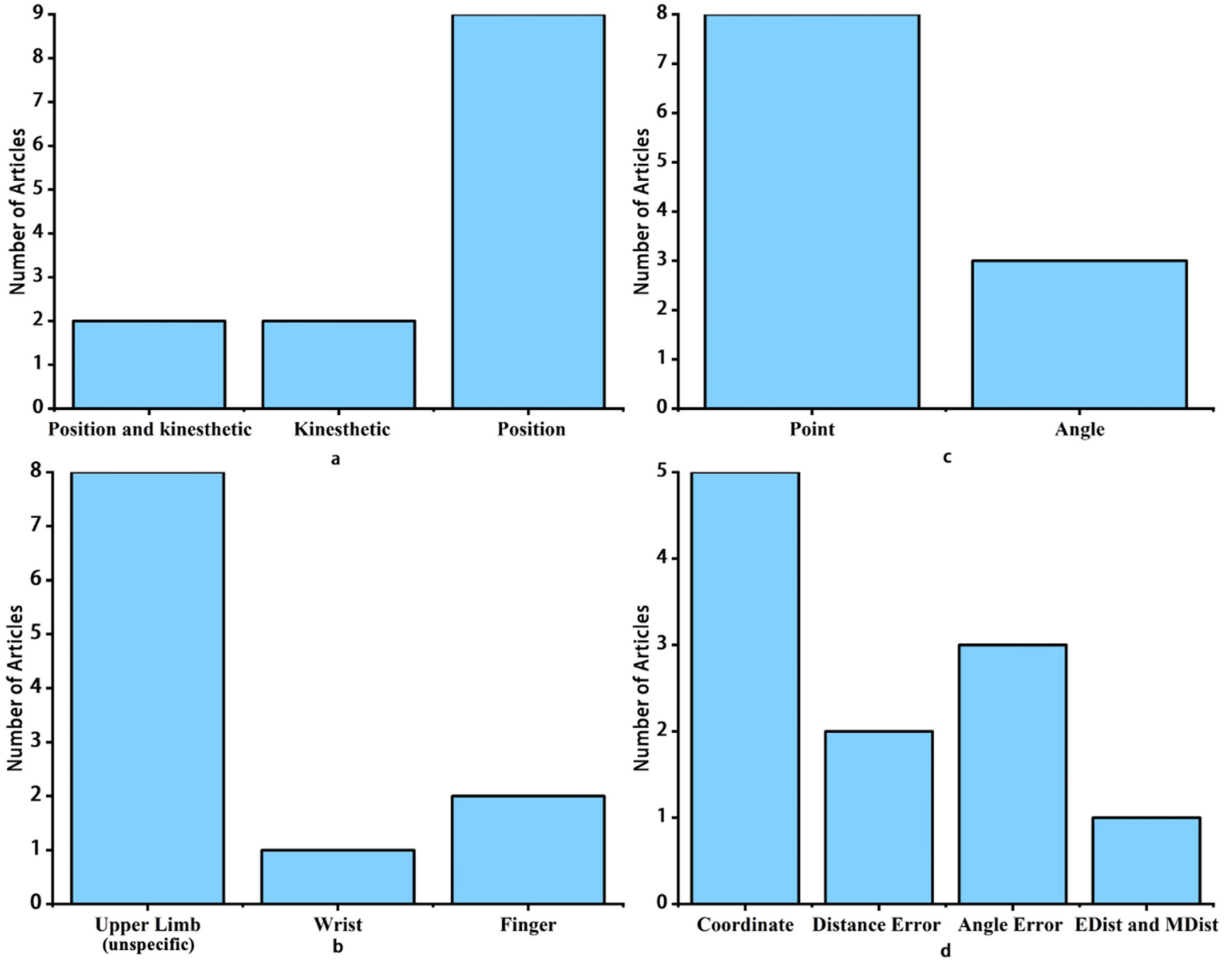
Overview of proprioceptive assessment components in stroke studies: **(a)** Types of proprioception assessed, **(b)** Locations of position sense assessment, **(c)** Matching targets used, and **(d)** Quantitative outcomes of position sense assessments. EDist, Euclidean Distance; MDist: Mahalanobis Distance. Remark: **(a)** presents statistics for all included studies, while **(b-d)** only include studies measuring position sense.

A total of 2,646 articles were retrieved from the database searches, of which 13 articles were ultimately included. Figure 1 details the screening process of study inclusion and exclusion. The characteristics of included studies are summarized in Supplementary Table S2. Figure 2a summarizes the types of proprioception assessed in these studies. Figure 2b summarizes the locations assessed for position sense. Figure 2c summarized the matching target. The quantitative outcomes of position sense assessments were summarized in Figure 2d.

### 3.2 Quality assessment results

The results of the quality assessment for included studies are shown in Supplementary Table S3. Overall, no study rated as having a low risk of bias across all domains. Eight studies (61.5%) were classified as having a moderate risk of bias, primarily due to concerns in patient selection (e.g., exclusion of severe cases, age mismatch, or device dependency) and reference standard (e.g., high risk in 8/13 studies due to lack of blinding or inappropriate reference standards). Two studies (28, 29) were rated as having a high risk of bias, with issues in patient selection (e.g., limited workspace, exclusion of distal joints) and index test (e.g., moderate risk due to lack of blinding). Applicability concerns were moderate to high in most studies, often due to strict inclusion criteria (e.g., exclusion of severe motor/cognitive deficits, focus on specific planes of movement, or age mismatch with controls). These findings suggest that the overall quality of the included studies was moderate, but caution should be exercised when interpreting results from studies with high or moderate risk of bias, particularly in the domains of patient selection and reference standards.

### 3.3 The method of assessment

#### 3.3.1 Position sense

**Figure 3:**
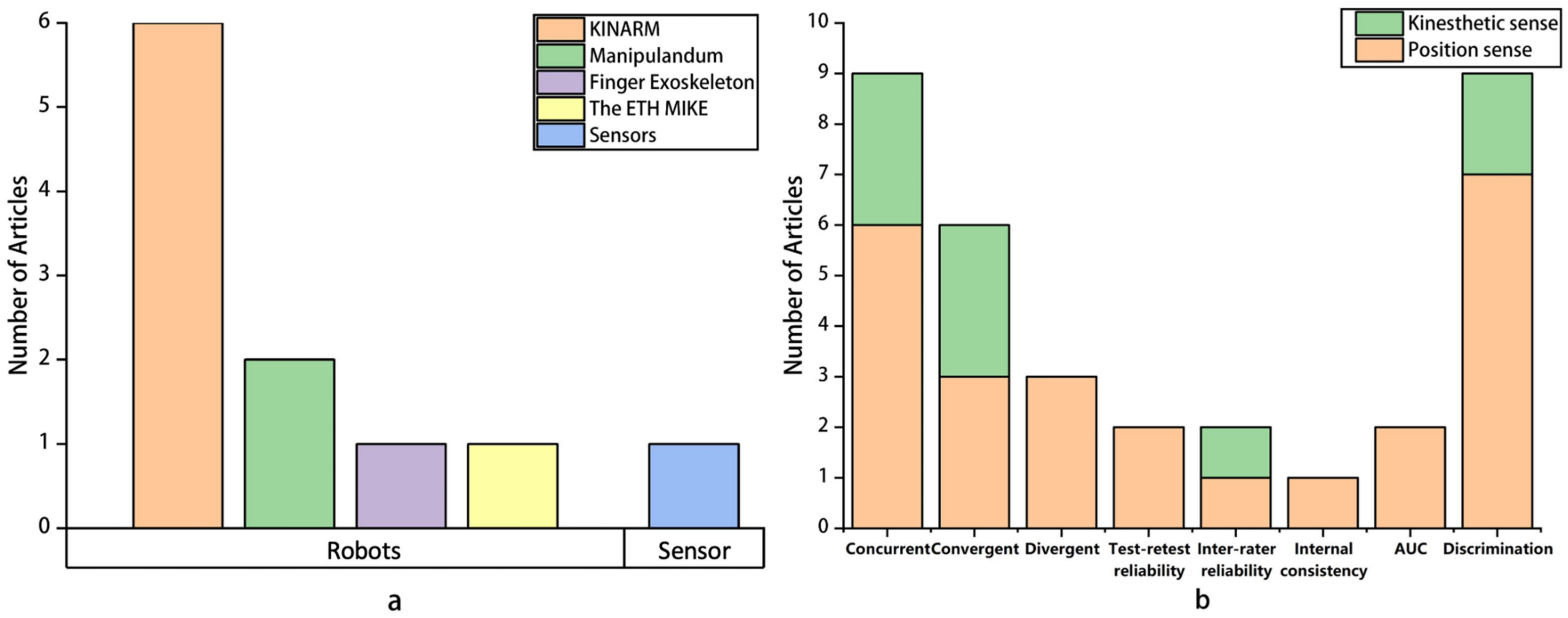
Equipment for position sense assessment and psychometric properties of assessment: **(a)** number of studies by equipment for position sense assessment, **(b)** number of studies by psychometric property in proprioceptive assessments. AUC, Area Under The Curve. Remark: Two articles investigated the concurrent and convergent validity of both kinesthetic sense and position sense; thus, in **(b)**, each of them is counted as two studies in the statistics.

Technologies used varied in terms of the time required, cost of equipment, complexity of operation, and number of factors potentially influencing the assessment results. A summary of currently applied technologies is shown in Figure 3a. Most studies utilized robot-based technologies (*n* = 10), with only one study employing sensors. For robot-assisted assessments, the majority of studies used the KINARM Exoskeleton (*n* = 6). The use of this device involves sitting in a wheelchair base with the arms supported by arm troughs to counteract gravity while performing matching tasks. Two studies used the manipulandum robotic device, which involves grasping a distal handle for matching tasks without the upper arm making contact with the machine. Robots used to assess the position sense of fingers and wrists separately fix adjacent parts of the joint to control the range of motion. The ETH MIKE is a device that controls finger movements using a machine, with matching tasks displayed on a screen using a simple gauge with a red indicator. For sensor-based assessments, a sensor is placed on the palm, and matching tasks are performed on a tabletop.

Locations assessed for position sense were also summarized, as different parts of the body engage in daily activities in various ways and may pose greater proprioceptive challenges than others when farther from the trunk. Figure 3b provides a summary of the locations assessed for position sense. Most articles comprehensively examined the entire upper limb, involving the shoulder, elbow, wrist, and hand (*n* = 8), one article assessed wrist position sense, and two articles evaluated finger position sense.

The method of matching is also an area of interest. Different matching methods have various factors that can influence assessment results, making some methods unsuitable for stroke patients with certain complications. As shown in Table 1, most newly developed assessments involve active mirror matching (*n* = 8), with only one study conducting passive unilateral matching. Additionally, two studies on finger position sense assessment employed unique matching methods: one involved the passive matching of the index and middle finger alignment (30), and the other used a simple gauge with a red indicator to match the finger position (19).

**Table 1:** Assessment method and psychometric properties.

Study	Type of proprioception	Equipment	Time (minutes)	Location	Matching target	Type of matching	Visual participation	Outcome	Psychometric properties
Kenzie et al. (37)	Position sense and kinesthetic sense	KINARM Exoskeleton	14–16	UL	Point and concurrent movement	Mirror-match; Active (Both: P → NP, matching)	No	EDist and MDist	Concurrent: TLT, r = 0.47–0.51 (*p* < 0.001) Convergent: CMSA, r = −0.50–−0.58; FIM, r = −0.40–−0.45
Semrau et al. (35)	Position sense and kinesthetic sense	KINARM Exoskeleton	NR	UL	Point and concurrent movement	Mirror-match; Active (Both: P → NP, matching)	No	Position: Coordinate Kinesthetic sense: IDE, PLR, RL, SLR	Concurrent: TLT, all significant Convergent: FIM, CMSA, and PPB all show significant
Semrau et al. (36)	Kinesthetic sense	KINARM Exoskeleton	NR	UL	Concurrent movement	Mirror-match; Active (Both: P → NP, matching)	No	IDE, PLR, RL, SLR	Inter-rater: r = 0.69–0.95 Discrimination: Significantly different
Semrau et al. (34)	Kinesthetic sense	KINARM Exoskeleton	NR	UL	Concurrent movement	Mirror-match; Active (Both: P → NP, matching)	No	IDE, PLR, RL, PSR	Concurrent: TLT, half outcome significant Convergent: FIM, all show significant (*p* < 0.0063) Discrimination: Significantly different
Otaka et al. (33)	Position sense	KINARM Exoskeleton	20–30	UL	Point	Mirror-match; Active (Both: P → NP, matching)	No	Coordinate	Concurrent: TLT, Var_xy_ (*p* = 0.011, r = 0.40); Contr/Exp_xy_ (*p* < 0.001, r = −0.71); Shift (*p* = 0.093, r = 0.27)
Dukelow et al. (32)	Position sense	KINARM Exoskeleton	NR	UL	Point	Mirror-match; Active (Both: P → NP, matching)	No	Coordinate	Concurrent: TLT, Var_xy_ and Shift_xy_ significant Convergent: FIM, all show significant (*p* < 0.0013) Internal consistency: significant (*p* < 0.0014)
Dukeow et al. (17)	Position sense	KINARM Exoskeleton	3–6	UL	Point	Mirror-match; Active (Both: P → NP, matching)	No	Coordinate	Inter-rater: r = 0.70–0.86 Discrimination: Significantly different
Contu et al. (38)	Position sense	Manipulandum robotic device (H-Man robotic device)	<10	UL	Point	Ipsilateral; Passive (P only, no matching)	No	Distance Error	Discrimination: No significantly different
Cusmano et al. (31)	Position sense	Manipulandum robotic device (2-degree-of-freedom robotic device)	~20	UL	Point	Mirror-match; Active (Both: P → NP, matching)	No	Coordinate	Test–retest: ICC = 0.72–0.84 Discrimination: Significantly different
Leibowitz et al. (29)	Position sense	Sensors	~15	UL	Point	Mirror-match; Active (Both: P → NP, matching)	No	Distance Error	Concurrent: Up-or-Down, r = 0.647 (*p* < 0.01) Divergent: 0–3 scale, no significant Discrimination: Significantly different
Basteris et al. (28)	Position sense	WristBot	~8	Wrist	Angle	Mirror-match; Active (Both: P → NP, matching)	No	Absolute Error (Angle)	Discrimination: Significantly different
Ingemanson et al. (30)	Position sense	Finger robotic exoskeleton	2 (EH)	Finger	Angle	Matching of the index and middle finger alignment; Passive (P only, no matching)	No	Absolute Error (Angle)	Divergent: Not correlated with most motor assessments (BBT, NHPT, FT, and motor FMA arm assessments.) and other clinical tests (NIHSS); ARAT, r = −0.42 (*p* = 0.03). AUC: AUC = 0.883 Discrimination: Significantly different
Zbytniewska et al. (19)	Position sense	The ETH MIKE	13–14	Finger	Angle	Use a simple gauge with a red indicator to match the finger position (P only, no matching)	No	Absolute Error (Angle)	Concurrent: kUDT, r = −0.48 (*p* = 0.007) Divergent: BBT, r = −0.37; FMA, no significant correlation. Test–retest: ICC = 0.90 (0.88–0.91); Correlation = 0.74 (*p* < 0.001) AUC: AUC = 0.82–0.95 (*p* < 0.001) Discrimination: Significantly different

NR, Not reported; EH, each hand; UL, Upper Limb; Visual Participation, whether visual input was involved in the matching task (e.g., “without” indicates no visual input); Point (Coordinate-Based), a matching task where the target is a spatial position perceived as a point in space, recorded as coordinates; Joint Angle Matching, a matching task where the target is the shoulder and elbow joint angles, reflecting perceived joint position; Coordinate, spatial position of the hand recorded as coordinates; EDist, Euclidean distance; MDist, Mahalanobis distance; Both, Both limbs; P, Paretic limb; NP, Non-paretic limb; P → NP, Passive on paretic limb, active on non-paretic limb; matching, Paretic limb matches non-paretic limb (or vice versa); no matching, No matching between limbs; DE, Distance error; Coordinate, Three outcomes are calculated using the coordinates of the points including Contr/Expxy (Spatial contraction/expansion area), Varxy (distance Variability) and Shiftxy (Systematic shifts); IDE, Initial Direction Error; PLR, Path Length Ratio; RL, Response Latency; PSR, Peak Speed Ratio; Contr/Exp_xy_, Spatial contraction/expansion area; Var_xy_, distance Variability; Shift_xy_, Systematic shifts; TLT, Thumb Localizing Test; kUDT, The kinesthetic Up-Down Test; NSA, Nottingham Sensory Assessment; FIM, Functional Independence Measure; CMSA, Chedoke-McMaster Stroke Assessment; BBT, the Box and Block Test of Manual Dexterity; PPT, The Purdue Pegboard Test; FMA, Fugl-Meyer Assessment; ARAT, Action Research Arm Test; FT, Finger Tapping Test; NHPT, Nine Hole Peg Test; NIHSS, National Institutes of Health Stroke Scale; AUC, the Area Under the Curve; ICC, Intraclass Correlation Coefficient.

Additionally, the proprioceptive abilities required for different matching targets, their similarity to daily life, and how results are quantified also varied. As illustrated in Figure 2c, most studies used a distal hand point (Coordinate-Based) as the matching target (*n* = 8), while only a few used joint angles as the matching target (*n* = 3). The assessment of upper limb position sense is done by matching target points, with results based on the distance between the two points (Coordinate-Based). In studies that use plane coordinates to represent points (Coordinate-Based), parameters included distance variability, areas of spatial contraction/expansion, and systematic shifts (17, 31–36). One study utilized two common distance measurement methods, Euclidean Distance (EDist) and Mahalanobis Distance (MDist), to integrate these three parameters of position sense (37). Additionally, some studies quantified results solely based on the distance error between two points (Coordinate-Based) (29, 38). Assessments of wrist and finger proprioception are done by matching target angles, with results expressed in terms of absolute angular error. Figure 2d summarizes the quantitative outcomes of position sense assessments.

#### 3.3.2 Kinesthetic sense

In the studies related to assessing kinesthetic sense, the KINARM robotic exoskeleton was used to assess the entire upper limb, which involved the shoulder, elbow, wrist, and hand, with the hand’s spatial coordinates recorded as the position outcome to evaluate proprioception (34–37) (Table 1). Participants were asked to move their active arm to match the speed, direction, and amplitude of their passive arm as soon as they felt the robotic arm move their own. To quantify kinesthesia, four kinematic parameters were used to describe the nature of an individual’s proprioceptive impairments: Initial Direction Error (IDE, measuring accuracy of movement initiation), Path Length Ratio (PLR, indicating movement efficiency), Response Latency (RL, reflecting reaction time), and Peak Speed Ratio (PSR, assessing speed matching accuracy). These parameters are detailed in Supplementary Table S3. In one study EDist and MDist distance measures were used to integrate all the parameters in a positional and kinesthetic matching robotics task (37).

### 3.4 Psychometric properties

Figure 3b summarizes the psychometric properties examined in these studies. The studies placed significant emphasis on examining these properties, particularly concurrent validity, with seven of them addressing this aspect. Additionally, the majority of studies evaluated the responsiveness of the assessment tools (*n* = 9). However, only a few studies tested the reliability of their assessment tools.

#### 3.4.1 Position sense

*Validity*: A total of six position sense assessment studies investigated concurrent validity. The TLT, kUDT, and the 0–3 scale were applied as reference tests. Most studies demonstrated significant correlations, with correlation coefficients ranging from 0.40 to 0.71 (moderate to high correlations) (Table 1). Three studies involving the KINARM robot reported correlation coefficients approximately between-0.40 and-0.50 with the Functional Independence Measure (FIM), the Canadian Motor Skills Assessment (CMSA), and the General Physical Performance scores, indicating moderate to high correlations (Table 1). Three studies investigated divergent validity, with most results showing no significant correlation with assessments which not focus on proprioceptive measurement like the Fugl-Meyer Assessment (FMA). However, some studies found low but significant correlations, such as correlation coefficients of-0.42 with the Action Research Arm Test (ARAT) and-0.37 with the Box and Blocks Test (BBT) (Table 1).

*Reliability*: Two position sense assessment tools were evaluated for test–retest reliability, both demonstrating high reliability with ICCs ranging from 0.72 to 0.84 and 0.90 (Table 1). One study indicated that consistency was high among evaluators in the KINARM robot assessment, with inter-rater correlations (r) ranging from 0.70 to 0.86. Internal consistency in the KINARM robotic study by Dukelow et al. (32) was also considered significant (Table 1).

*Responsiveness*: Two studies on finger proprioceptive assessment evaluated the ability to distinguish using ROC curve analysis. Ingemanson et al. (30) reported an AUC of 0.883, while Zbytniewska et al. (19) reported AUCs of 0.82. These results demonstrated good discrimination. The other five studies simply compared whether there were significant differences between stroke patients and healthy individuals (Table 1). Most studies indicated significant differences in proprioception between stroke patients and healthy individuals, except for the study by Contu et al. (38), which found no significant differences (*p* = 0.46).

#### 3.4.2 Kinesthetic sense

*Validity*: Three kinesthetic sense assessment studies tested concurrent validity, with correlation coefficients between TLT and various parameters ranging from 0.47 to 0.48, indicating moderate to high correlations (Table 1). Three studies on kinesthetic assessments also validated convergent validity. These studies showed high correlation coefficients with the FIM (r = 0.44), the CMSA (r = 0.56), and the Purdue Pegboard test (Table 1). These findings suggest that the convergent validity of the KINARM Robotics Assessment is moderate to high. No kinesthetic sense assessments validated divergent validity (Table 1).

*Reliability*: Only one study assessed inter-rater reliability for kinesthetic assessments. The inter-rater reliability varied across different parameters, with correlations (r) ranging from 0.69 to 0.95 (Table 1).

*Responsiveness*: Two studies on kinesthetic assessment tools compared differences between healthy individuals and stroke patients, finding significant differences between the two groups (Table 1).

## 4 Discussion

This review provides a comprehensive synthesis of various methods for assessing proprioception in stroke patients using technologies based on a matching paradigm. We examined 13 studies, and the majority employed exoskeleton robots, with mirror-matching methods being the most common. In terms of psychometric properties, these studies demonstrate moderate to high reliability, including test–retest reliability and inter-rater consistency. Overall validity was considered to be good based on moderate to high correlations with existing relevant clinical proprioceptive (TLT and kUDT) and other related measurements (FIM), and no correlation with unrelated assessments (FMA).

The psychometric properties reported in these studies demonstrate that these assessment tools can serve as reliable quantitative methods for evaluating proprioceptive deficits in the upper limbs following stroke. The proprioceptive assessment tools exhibit a higher degree of reliability than those currently used in clinical practice (19), making them more dependable for clinical applications. However, the validity of these proprioceptive assessment tools is only moderately correlated with clinical assessment tools. Nevertheless, given the absence of a gold standard and the low validity of the clinical assessment tools currently in use (10, 15, 39), these tools are believed to accurately assess the degree of proprioceptive dysfunction. The lower validity may be due to the poor sensitivity and psychometric quality of previous clinical assessment tools, whereas newer tools exhibit higher sensitivity, thereby resulting in a lower overall correlation (19). A key challenge in this field is the lack of a universally accepted gold standard, as traditional tests like the TLT, while widely used, are affected by subjectivity and limited sensitivity. It may cause the underestimation of the true validity of technology-assisted methods. Further research is needed to establish more robust validity as the field evolves.

The methods used to evaluate the psychometric properties of proprioceptive assessments in these studies were somewhat inconsistent. Due to the lack of a gold standard for proprioceptive assessment tools, concurrent validity was assessed by comparing them with common clinical tests. Studies have shown that the TLT is more sensitive and has higher concurrent validity than other tests like limb localization (40), making it a recommended comparison for new tools. In addition, convergent validity was often evaluated using scales that assess functional activities, such as the FIM scale for activities of daily living (41), which correlates with proprioceptive dysfunction (9, 32). The studies included in this review show significant correlations between proprioceptive assessment tools and the FIM scale (32, 34, 35, 37), suggesting newer tools could be used to determine convergent validity. However, while these correlations provide some evidence of concurrent validity, the broader application of this approach in proprioceptive assessment systems warrants further discussion. Héroux et al. (42) proposed a novel framework for assessing proprioception, by distinguishing between low-level judgments (e.g., detecting limb position) and high-level judgments (e.g., integrating multiple spatial references) (42). This framework underscores the need for assessment tools to capture both basic and advanced proprioceptive functions, which may not be fully addressed by traditional clinical tests like TLT or functional scales like FIM. Similarly, Krewer et al. (43) emphasized that proprioception encompasses multiple aspects, such as threshold detection versus supra-threshold discrimination, and caution against using comparison tools that do not align with the specific construct being assessed (43). For instance, while the FIM scale is valuable for evaluating functional outcomes, it may not fully capture sensory-specific proprioceptive constructs, potentially limiting its utility as a reference for concurrent validity in certain contexts.

Conversely, divergent validity examines whether unrelated concepts remain uncorrelated. Within the included studies, divergent validity was examined for only a limited number of position sense assessment tools, typically through comparison with motor function scales. As theoretically predicted, these analyses revealed no significant associations, confirming that position sense deficits are conceptually distinct from motor impairments (32, 44). For example, a study showed no significant correlation between position sense and FMA, an observation-based assessment for motor impairment (45). In contrast, the BBT, which evaluates dexterity (46), and the ARAT, which assesses coordination, dexterity, and function (47), showed weak correlation. As position sense affects activity performance, the lack of correlation may be due to the fact that these two assessments evaluate motor function through activity rather than standardized tasks (44). Therefore, the FMA may be better suited for testing divergent validity. Given the absence of a gold standard, this multi-faceted approach—including convergent and divergent validity—strengthens the evidence base for these tools by providing a comprehensive evaluation beyond concurrent validity alone. Moreover, the challenges in establishing concurrent validity, as highlighted by Héroux et al. (42) and Krewer et al. (43), suggest the need for standardized protocols and consensus-based theoretical frameworks in order to guide future assessments (42, 43).

Furthermore, in evaluating responsiveness, many studies only compared results between patients with stroke and healthy controls (17, 29, 31, 34). However, it is also important to use ROC to determine the ability of assessments to distinguish between patients with stroke and healthy control subjects.

These assessment methods have some limitations in providing a comprehensive evaluation of proprioception. The matching paradigm evaluates the following proprioceptive pathway: signals such as the position, velocity, and force of the limbs activate mechanoreceptors in the skeletal muscles, specifically muscle spindles and Golgi tendon organs. The dorsal column-medial lemniscus (DCML) pathway serves as the primary conduit for transmitting proprioceptive signals from peripheral receptors to the thalamus and primary somatosensory cortex (S1), enabling conscious perception of limb position. However, contemporary lesion and neuroimaging studies demonstrate that proprioception relies on extended networks: cortical regions mediate distinct functional roles through multisensory integration and predictive processing. For example, the temporoparietal junction integrates proprioceptive input with vestibular and visual cues to maintain coherent body representation (48), while the supramarginal gyrus maps proprioceptive information onto spatial coordinates for action planning (37). The superior temporal gyrus refines sensorimotor predictions by comparing expected versus actual limb positions (48), and the parietal operculum encodes limb position relative to external objects during goal-directed movements (25). Subcortically, the thalamus prioritizes DCML-derived signals through its ventral posterior nuclei, whereas cerebellar-thalamocortical circuits dynamically adjust motor outputs based on proprioceptive error signals (2). This distributed processing explains why traditional matching tasks cannot localize lesions to specific anatomical nodes—a deficit in spatial mapping (e.g., supramarginal gyrus damage) may mimic DCML dysfunction despite intact signal transmission. Future assessments should combine kinematic measures with functional neuroimaging to disentangle contributions of the core DCML pathway from higher-order integrative regions.

Moreover, different matching methods are influenced by several factors. Mirror-matching involves both sides of the upper limb, making it challenging to locate the affected side and requiring inter-hemispheric communication during the assessment, which may be difficult for some patients with stroke (21, 49). Ipsilateral matching requires passive matching due to motor impairments on the affected side, eliminating the need for interhemispheric information transfer (21). However, this method is limited in cases where bilateral motor deficits or memory impairments are present in stroke patients (21). Image matching effectively reduces confusion from interhemispheric transfer and motor deficits but does not provide information on kinesthetic impairment. Additionally, visual errors, such as parallax, could distort proprioceptive testing results (50, 51).

Furthermore, different matching targets require varying levels of proprioceptive ability and have different degrees of relevance to daily functional activities. Matching targets are classified into point targets in external personal space (hand position sense) and simple joint targets in internal joint space (limb position sense) (52). Although hand position sense and limb position sense are inherently related due to the anatomical connection between the hand and arm, they involve distinct proprioceptive processes. Hand position sense relies more on the integration of multisensory inputs (e.g., visual and tactile cues) to accurately locate the hand in external space, which is essential for performing precise and skilled bimanual tasks in daily life. In contrast, limb position sense primarily depends on joint angle perception and is more relevant for gross motor control. Previous studies suggest that point targets, which require hand position sense, demand a higher level of multisensory integration compared to joint targets, which rely on limb position sense (53). From a functional perspective, hand position sense is considered to be more complex and critical for performing skilled bimanual tasks in daily life compared to limb position sense.

When choosing technologies for the assessment of proprioception, it is advisable to choose techniques that provide the least amount of additional information such as vision and pressure sense. Because the tactile and pressure feedback from the robotic device’s arm may introduce extra sensory information that could aid in localization (17).

In the future, the development of new proprioceptive assessment tools should aim to address current limitations while focusing on the following aspects for improvement. First, in terms of the modality used, the integration of more affordable and portable solutions, such as virtual reality (VR), could significantly reduce costs while maintaining or even enhancing functionality. Advances in VR technology for simulating complex environments have improved its accessibility. Second, in terms of the assessment method, current research primarily focuses on proprioception in a horizontal plane. Future tools should expand evaluations to be inclusive of three-dimensional (3D) space or vertical planes to be more reflective of proprioception during daily activities. Compared to the use of robots, cost-effective technologies like VR can facilitate the transition to 3D assessment while obviating additional expenses, as these systems are inherently designed to operate in 3D environments. When evaluating the psychometric properties of these assessment tools, it is important to include participants from the intended user group. Many tools intended for patients with stroke have only been tested in healthy individuals, thereby limiting their generalizability of the psychometric measurement properties being reported (22, 54–56). Including the target population is crucial for determining clinical applicability (26). Additionally, as proprioception may decline with age (57), comparisons should involve age-matched healthy control groups.

## 5 Limitations of the study

This scoping review specifically examined the matching paradigm—the most frequently used technology-assisted method for proprioceptive assessment. As such, alternative technology-assisted paradigms (e.g., discrimination tasks) were not considered in this review.

## 6 Conclusion

In this review, proprioceptive assessment tools based on new technologies that utilize matching paradigms demonstrated high reliability and moderate validity. The primary technology employed was robotics using a mirror-matching approach. However, some assessments could not identify which side of the body sustained proprioceptive damage, as well as disparities in interhemispheric communication and motor function may potentially affect the assessment results. It is crucial to develop assessment protocols that offer a more thorough evaluation of proprioception. Additionally, future studies may consider using more portable technologies for assessing proprioception in three-dimensional space.
