# Supplementary material for: Psychometric properties of technology-assisted matching paradigms in post-stroke upper limb proprioceptive assessment: a scoping review
Source: Front Neurol. 2025 Jun 26;16:1556111. doi: 10.3389/fneur.2025.1556111 (PMC12243609; doi:10.3389/fneur.2025.1556111)
Supplement: Supplementary file 1 [file Supplementary_file_1.docx]

**Supplementary materials**

Psychometric Properties of Technology-Assisted Matching Paradigms in Post-stroke Upper Limb Proprioceptive Assessment: A Scoping Review

**Table S1. Different types of validity**

| **Validity Type** | **Definition** | **Position Sense Example** | **Kinesthetic Sense Example** |
| --- | --- | --- | --- |
| Concurrent Validity | Comparison with an established reference measure. | Position sense assessments were compared with TLT (Dukelow et al., 2012; Kenzie et al., 2017; Otaka et al., 2020; Semrau et al., 2015) and the 'up-or-down' test (Leibowitz et al., 2008; Zbytniewska et al., 2021). | Kinesthetic sense assessments compared with TLT parameters (Semrau et al., 2015). |
| Convergent Validity | Correlation with tests of the same construct. | KINARM robot position sense assessment correlated with FIM, CMSA, PPB (Semrau et al., 2015). | KINARM robot kinesthetic sense assessment correlated with FIM, CMSA, and PPB (Semrau et al., 2015). |
| Divergent Validity | Lack of correlation with tests of different constructs. | Position sense assessments have no correlations with most motor assessments (BBT, NHPT, FT, and motor FM arm assessments.) and other clinical tests (NIHSS) (Ingemanson et al., 2019). | Not Reported |

| Table S2. Basic information on subject | | | | | | | |
| --- | --- | --- | --- | --- | --- | --- | --- |
| Study | Proprioception (body part) | Group size | Age (years) | Gender (female/male) | Handedness (R, L, Mixed) | Post-stroke | Hemisphere Lesion (R/L/B) |
| Semrau et al., 2015 | Position sense and kinesthetic sense (upper limb) | Stroke (n=116) | NR | NR | NR | 10.6 ± 6.6 days  45.2 ± 5.9 days  90.0 ± 11.3 days  188.5 ± 15.5 days | NR |
| Kenzie et al., 2017 | Position sense and kinesthetic sense (upper limb) | Stroke (n=285) | 59.5 ± 14.7 (L)  61.2 ± 14.6 (R)  60.6 ± 14.6 (total) | 41/ 74 (L)  51, 119 (R)  92/193 (total) | 104, 10, 1 (L)  160, 8, 2 (R)  264, 18, 3 (total) | 12 ± 18 days (LH)  12 ± 12 days (RH)  12 ± 15 days (total) | 115/170 |
| Semrau et al., 2013 | Kinesthetic sense (upper limb) | Stroke (n=113)  HC (n=74) | RH: 64 (24-89)  LH: 63 (18-89)  HC: 61 (18 - 88) | 17/45 (RH)  22/29 (LH)  37/37 (HC) | 58, 1, 3 (RH)  44, 3, 4 (LH)  64, 10, 0 (HC) | RH:11 (1-48) days  LH:9 (2-51) days | 62/51 |
| Semrau et al., 2017 | Kinesthetic sense (upper limb) | Stroke (n=15)  HC (n=25) | 54.5±13.6 (stroke)  38.3±13.0 (HC) | 3/15 (stroke)  17/8 (HC) | 14, 1, 0 (stroke)  22, 3, 0 (HC) | 62.4 ± 63.4 days | 5/8/2 |
| Dukelow et al., 2010 | Position sense (upper limb) | LH (n=24)  RH (n=21)  HC (n=65) | LH: 66 (29-84)  RH: 66 (22-90)  HC: 63 (22-93) | 8/16 (LH)  12/9 (RH)  40/25 (HC) | 24, 0, 0 (LH)  18, 0, 2 (RH)  59, 2, 4 (HC) | LH: 31 (15-76) days  RH: 30 (11-206) days | 21/24 |
| Dukelow et al., 2012 | Position sense (upper limb) | RH (n=46)  LH (n=54)  HC (n=231) | RH: 64 (22-90)  LH: 62 (21-84) | 21/25 (RH)  22/32 (LH)  123/108 (HC) | 41, 1, 4 (RH)  41, 7, 6 (LH)  208, 14, 9 (HC) | RH: 25 (5-75) days  LH: 31 (6-81) days | 46/54 |
| Otaka et al., 2020 | Position sense (upper limb) | Stroke (n=40) | 47.6 ± 12.2 (stroke) | 15/25 (stroke) | NR | 486.5 (164-6456) days | NR |
| Leibowitz et al., 2008 | Position sense (upper limb) | Stroke (n=22)  HC (n=9) | Stroke: 29-79 (62.1)  HC: 28-80 (53) | 12/10 (stroke)  4/5 (HC) | All subjects, in both groups, were right-handed. | 3.5-19.6 week | 11/11 |
| Meng et al., 2014 | Position sense (upper limb) | Stroke (n=9)  HC (n=15) | 61.8 ± 1.3 (stroke)  32.7 ± 11.5 (HC) | 1/8 (stroke)  8/7 (HC) | NR | 29.8 ± 38.9 months | 6/3 |
| Contu et al., 2017 | Position sense (upper limb) | Stroke (n=9)  HC (n=9) | 53.7 ± 7.3 (stroke)  54.8 ± 3.9 (HC) | 4/5 (stroke)  6/3 (HC) | 8, 1, 0 (stroke)  9, 0, 0 (HC) | 14.4 ± 8.4 months | 2/7 |
| Basteris et al., 2018 | Position sense (wrist) | Stroke (n=10)  HC (n=10) | 54 ± 12.1 (stroke)  39 ± 15 (HC) | 1/9 (stroke)  5/5 (HC) | 10, 0, 0 (HC) | 18.70 ± 12.50 days | 6/4 |
| Ingemanson et al., 2019 | Position sense (finger) | Stroke (n=27)  HC (n=25) | 58 ± 14 (stroke)  44.5 ± 9.4 (12 middle-aged HCs)  73.3 ± 6.8 (13 older HCs) | 8/19 (stroke)  9/3 (12 middle-aged HCs)  5/8 (13 older HCs) | 24, 3, 0 (stroke) | chronic stroke | 14/13 |
| Zbytniewska et al., 2021 | Position sense (finger) | Stroke (n=30)  HC (n=31) | 64.50 ± 14.02 (stroke)  66.87 ± 7.92 (HC) | 11/19 (stroke)  11/20 (HC) | 26, 4, 0 (stroke)  31, 0, 0 (HC) | 8.17 ± 4.56 weeks | 18/12 |

Abbreviations: LH: Left Hemisphere; RH: Right Hemisphere; R: Right; L: Left; B: Bilateral; HC: Health control; NR: Not Reported

**Table S3. Quality assessment result.**

| **Study** | **Patient Selection** | **Index Test** | **Reference Standard** | **Flow and Timing** | **Overall Risk of Bias** |
| --- | --- | --- | --- | --- | --- |
| Semrau et al., 2015 | Moderate | Low | High | Low | Moderate |
| Kenzie et al., 2017 | Moderate | Low | Moderate | Moderate | Moderate |
| Semrau et al., 2013 | Moderate | Low | High | Low | Moderate |
| Semrau et al., 2017 | Moderate | Low | High | Low | Moderate |
| Dukelow et al., 2010 | Moderate | Low | Moderate | Low | Moderate |
| Dukelow et al., 2012 | Moderate | Low | High | Low | Moderate |
| Otaka et al., 2020 | Moderate | Low | High | Low | Moderate |
| Leibowitz et al., 2008 | Moderate | Low | High | Low | Moderate |
| Meng et al., 2014 | Moderate | Low | Moderate | Low | Moderate |
| Contu et al., 2017 | High | Moderate | High | Moderate | High |
| Basteris et al., 2018 | Moderate | Low | High | Low | Moderate |
| Ingemanson et al., 2019 | Moderate | Low | High | Low | Moderate |
| Zbytniewska et al., 2021 | Moderate | Low | Moderate | Low | Moderate |

**References**

Dukelow, S. P., Herter, T. M., Bagg, S. D., & Scott, S. H. (2012). The independence of deficits in position sense and visually guided reaching following stroke. *J Neuroeng Rehabil*, *9*, 72. <https://doi.org/10.1186/1743-0003-9-72>

Ingemanson, M. L., Rowe, J. R., Chan, V., Riley, J., Wolbrecht, E. T., Reinkensmeyer, D. J., & Cramer, S. C. (2019). Neural Correlates of Passive Position Finger Sense After Stroke. *Neurorehabil Neural Repair*, *33*(9), 740-750. <https://doi.org/10.1177/1545968319862556>

Kenzie, J. M., Semrau, J. A., Hill, M. D., Scott, S. H., & Dukelow, S. P. (2017). A composite robotic-based measure of upper limb proprioception. *J Neuroeng Rehabil*, *14*(1), 114. <https://doi.org/10.1186/s12984-017-0329-8>

Leibowitz, N., Levy, N., Weingarten, S., Grinberg, Y., Karniel, A., Sacher, Y., Serfaty, C., & Soroker, N. (2008). Automated measurement of proprioception following stroke. *Disabil Rehabil*, *30*(24), 1829-1836. <https://doi.org/10.1080/09638280701640145>

Otaka, E., Otaka, Y., Kasuga, S., Nishimoto, A., Yamazaki, K., Kawakami, M., Ushiba, J., & Liu, M. (2020). Reliability of the thumb localizing test and its validity against quantitative measures with a robotic device in patients with hemiparetic stroke. *PLoS One*, *15*(7), e0236437. <https://doi.org/10.1371/journal.pone.0236437>

Semrau, J. A., Herter, T. M., Scott, S. H., & Dukelow, S. P. (2015). Examining Differences in Patterns of Sensory and Motor Recovery After Stroke With Robotics. *Stroke*, *46*(12), 3459-3469. <https://doi.org/10.1161/strokeaha.115.010750>

Zbytniewska, M., Kanzler, C. M., Jordan, L., Salzmann, C., Liepert, J., Lambercy, O., & Gassert, R. (2021). Reliable and valid robot-assisted assessments of hand proprioceptive, motor and sensorimotor impairments after stroke. *J Neuroeng Rehabil*, *18*(1), 115. <https://doi.org/10.1186/s12984-021-00904-5>
